# Poly[di-μ-chlorido-μ-(1,2,3,9-tetra­hydro­pyrrolo­[2,1-*b*]quinazolin-9-one-κ^2^ *N*:*O*)-mercury(II)]

**DOI:** 10.1107/S1600536811022471

**Published:** 2011-06-18

**Authors:** Kambarali K. Turgunov, Yutian Wang, Ulli Englert, Khusnutdin M. Shakhidoyatov

**Affiliations:** a S. Yunusov Institute of the Chemistry of Plant Substances, Academy of Sciences of Uzbekistan, Mirzo Ulugbek Str. 77, Tashkent 100170, Uzbekistan; b Institute of Inorganic Chemistry, RWTH Aachen University, Landoltweg 1, D-52056 Aachen, Germany

## Abstract

In the crystal structure of the title two-dimensional network, [HgCl_2_(C_11_H_10_N_2_O)]_*n*_, the asymmetric unit consists of HgCl_2_ dumbbells and one mol­ecule of the quinazoline unit. Pseudo-octa­hedrally coordinated Hg^II^ cations are chloride-bridged *via* a crystallographic inversion centre leading to different Hg—Cl bonds (short and long) and linked by other Cl atoms *via* translation along the *a* axis. The quinazoline ligands connect the Hg—Cl—Hg—Cl chains by N and O atoms along the *b* axis, forming the two-dimensional network structure. The crystal structure is stabilized by weak non-classical C—H⋯Cl hydrogen bonds and aromatic π–π stacking inter­actions [centroid–centroid distances = 3.942 (4) and 3.621 (4) Å].

## Related literature

For the synthesis of the ligand, see: Chatterjee & Ganguly (1968 ▶). For the crystal structure of the ligand, see: Turgunov *et al.* (1995 ▶). For the crystal structure of the pure octa­hedral Hg^II^ ion and halide-bridged complex, see: Hu *et al.* (2007 ▶). For the crystal structure of a Hg^II^ complex with asymmetric Hg—Cl bonds, see: Batten *et al.* (2002 ▶); Hu *et al.* (2007 ▶); Merkens *et al.* (2010 ▶). For a general review of halide-bridged chain and crosslinking polymers, see: Englert (2010 ▶).
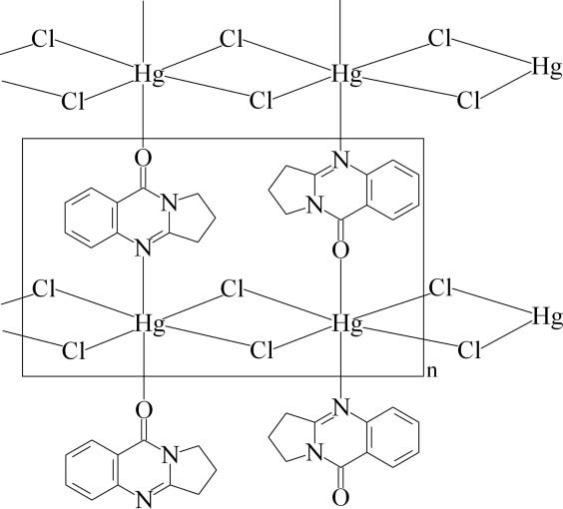


## Experimental

### 

#### Crystal data

[HgCl_2_(C_11_H_10_N_2_O)]
*M* *_r_* = 457.70
Monoclinic, 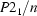

*a* = 7.7275 (11) Å
*b* = 9.4705 (13) Å
*c* = 16.729 (2) Å
β = 101.416 (2)°
*V* = 1200.1 (3) Å^3^
*Z* = 4
Mo *K*α radiation
μ = 13.25 mm^−1^
*T* = 130 K
0.21 × 0.09 × 0.08 mm

#### Data collection

Bruker SMART APEX diffractometer
Absorption correction: multi-scan (*SADABS*; Sheldrick, 1996 ▶) *T*_min_ = 0.167, *T*_max_ = 0.417
13274 measured reflections
3014 independent reflections
2620 reflections with *I* > 2σ(*I*)
*R*_int_ = 0.041

#### Refinement

*R*[*F* ^2^ > 2σ(*F* ^2^)] = 0.042
*wR*(*F* ^2^) = 0.099
*S* = 1.20
3014 reflections
154 parameters
H-atom parameters constrained
Δρ_max_ = 6.57 e Å^−3^
Δρ_min_ = −1.26 e Å^−3^

### 

Data collection: *SMART* *APEX* (Bruker, 2000 ▶); cell refinement: *SAINT-Plus* (Bruker, 1999 ▶); data reduction: *SAINT-Plus*; program(s) used to solve structure: *SHELXS97* (Sheldrick, 2008 ▶); program(s) used to refine structure: *SHELXL97* (Sheldrick, 2008 ▶); molecular graphics: *XP* (Bruker, 1998 ▶); software used to prepare material for publication: *publCIF* (Westrip, 2010 ▶).

## Supplementary Material

Crystal structure: contains datablock(s) I, global. DOI: 10.1107/S1600536811022471/si2358sup1.cif

Structure factors: contains datablock(s) I. DOI: 10.1107/S1600536811022471/si2358Isup2.hkl

Additional supplementary materials:  crystallographic information; 3D view; checkCIF report

## Figures and Tables

**Table 1:** Selected bond lengths (Å)

Hg1—Cl1	2.3258 (16)
Hg1—Cl2	2.3302 (16)
Hg1—Cl1^i^	3.1301 (16)
Hg1—Cl2^ii^	3.0416 (16)
Hg1—O1^iii^	2.775 (6)
Hg1—N1	2.649 (6)

Symmetry codes: (i) 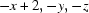; (ii) 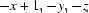; (iii) 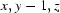.

**Table 2:** Hydrogen-bond geometry (Å, °)

*D*—H⋯*A*	*D*—H	H⋯*A*	*D*⋯*A*	*D*—H⋯*A*
C5—H5*A*⋯Cl1^iv^	0.93	2.81	3.630 (8)	147
C10—H10*B*⋯Cl2^v^	0.97	2.76	3.724 (9)	171

Symmetry codes: (iv) 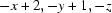; (v) 
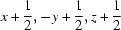
.

**Fig. 1.**
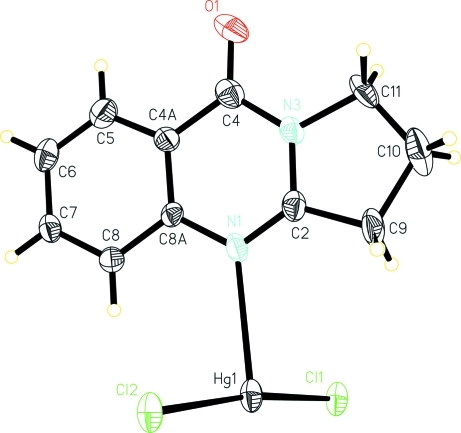
The asymmetric unit of the title coordination polymer (50% displacement ellipsoids).

**Fig. 2.**
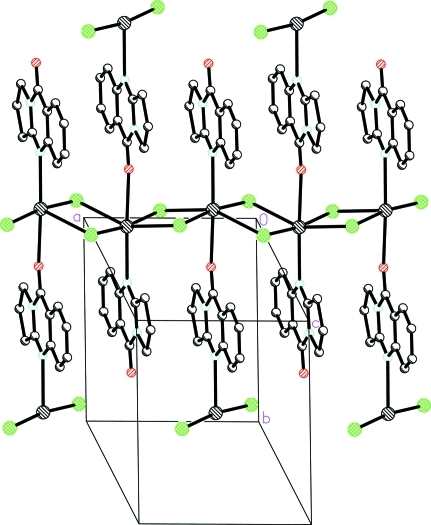
View of a single two-dimensional sheet. (Hydrogen atoms are omitted for clarity).

## supplementary crystallographic information

### Comment

The title compound represents the first crystal structure of a complex with the heterocyclic ligand 1,2,3,9-tetrahydropyrrolo(2,1 - b)quinazolin-9-one; the uncoordinated organic molecule has been reported by Turgunov *et al.* (1995).

The asymmetric unit contains a slightly bent HgCl_2_ moiety (Cl1—Hg1—Cl2 = 171.44 (6)°) and one ligand molecule (Fig.1) In the crystal each Hg^II^ cation is coordinated by four bridging chlorido ligands in the equatorial plane; one *N*- and one *O*-connected organic ligand occupy the axial positions of a distorted octahedron. The Hg—Cl bonds are asymmetric with two short and two longer distances (Table 1).

The bridging chlorido ligands form zigzag Hg—Cl—Hg—Cl ring chains in the direction of the shortest lattice parameter. Strongly asymmetric halide bridges are well established structural features in the coordination chemistry of divalent mercury (Batten *et al.*, 2002; Hu *et al.*, 2007; Merkens *et al.*, 2010; Englert, 2010).

The chlorido-bridged Hg···Hg distances amount to 3.9342 (7) and 3.9442 (7) Å. As the result of halide bridging in the [100] and bridging of the ditopic organic ligand in the [010] direction, an overall two-dimensional sheet is formed which is depicted in Fig. 2.

The observed structure is stabilized by weak C—H···Cl hydrogen bonds (Table 2). Cooperative π–π stacking interactions between neighbouring quinazolone ring systems also contribute to the stability of this layer structure (*Cg*1···*Cg*1^i^=3.942 (4) *Cg*1···*Cg*2^i^=3.621 (4) Å, where *Cg*1 represents the centroid of the pyrimidinone and *Cg*2 that of the benzo ring centroid, (i): 2 - *x*,1 - *y*,-*z*).

The ligand molecule is essentially planar with a maximum deviation of 0.044 (7) Å for atom C10 and an r.m.s. deviation of 0.015 Å.

### Experimental

A solution of 27.15 mg (0.1 mmol) of mercury (II) chloride in 2 ml water was added to a solution of 18.62 mg (0.1 mmol) of 1,2,3,9-tetrahydropyrrolo(2,1 - b)quinazolin-9-one in 2 ml acetone. The solution was allowed for slow evaporation at the room temperature. Colourless needle shaped crystals were obtained after several days.

### Refinement

Carbon-bound H atoms were positioned geometrically and treated as riding on their C atoms, with C—H distances of 0.93 Å (aromatic) and 0.97 Å (CH_2_) and were refined with *U*_iso_(H)=1.2Ueq(C). After completion of the structure model, a difference Fourier synthesis resulted in a local maximum closer than 0.8 Å to Hg1.

### Crystal data

**Table:**

[HgCl_2_(C_11_H_10_N_2_O)]	*F*(000) = 848
*M**_r_* = 457.70	*D*_x_ = 2.533 Mg m^−^^3^
Monoclinic, *P*2_1_/*n*	Mo *K*α radiation, λ = 0.71073 Å
Hall symbol: -P 2yn	Cell parameters from 3815 reflections
*a* = 7.7275 (11) Å	θ = 2.2–28.3°
*b* = 9.4705 (13) Å	µ = 13.25 mm^−^^1^
*c* = 16.729 (2) Å	*T* = 130 K
β = 101.416 (2)°	Rod, colourless
*V* = 1200.1 (3) Å^3^	0.21 × 0.09 × 0.08 mm
*Z* = 4	

### Data collection

**Table:**

Bruker SMART APEX diffractometer	3014 independent reflections
Radiation source: fine-focus sealed tube	2620 reflections with *I* > 2σ(*I*)
graphite	*R*_int_ = 0.041
ω scans	θ_max_ = 28.5°, θ_min_ = 2.5°
Absorption correction: multi-scan (*SADABS*; Sheldrick, 1996)	*h* = −10→10
*T*_min_ = 0.167, *T*_max_ = 0.417	*k* = −12→12
13274 measured reflections	*l* = −22→22

### Refinement

**Table:**

Refinement on *F*^2^	Primary atom site location: structure-invariant direct methods
Least-squares matrix: full	Secondary atom site location: difference Fourier map
*R*[*F*^2^ > 2σ(*F*^2^)] = 0.042	Hydrogen site location: inferred from neighbouring sites
*wR*(*F*^2^) = 0.099	H-atom parameters constrained
*S* = 1.20	*w* = 1/[σ^2^(*F*_o_^2^) + (0.0432*P*)^2^ + 2.8193*P*] where *P* = (*F*_o_^2^ + 2*F*_c_^2^)/3
3014 reflections	(Δ/σ)_max_ = 0.001
154 parameters	Δρ_max_ = 6.57 e Å^−^^3^
0 restraints	Δρ_min_ = −1.26 e Å^−^^3^

### Special details

**Table:**

Geometry. All e.s.d.'s (except the e.s.d. in the dihedral angle between two l.s. planes) are estimated using the full covariance matrix. The cell e.s.d.'s are taken into account individually in the estimation of e.s.d.'s in distances, angles and torsion angles; correlations between e.s.d.'s in cell parameters are only used when they are defined by crystal symmetry. An approximate (isotropic) treatment of cell e.s.d.'s is used for estimating e.s.d.'s involving l.s. planes.
Refinement. Refinement of *F*^2^ against ALL reflections. The weighted *R*-factor *wR* and goodness of fit *S* are based on *F*^2^, conventional *R*-factors *R* are based on *F*, with *F* set to zero for negative *F*^2^. The threshold expression of *F*^2^ > σ(*F*^2^) is used only for calculating *R*-factors(gt) *etc*. and is not relevant to the choice of reflections for refinement. *R*-factors based on *F*^2^ are statistically about twice as large as those based on *F*, and *R*- factors based on ALL data will be even larger.

### Fractional atomic coordinates and isotropic or equivalent isotropic displacement parameters (Å^2^)

**Table:**

	*x*	*y*	*z*	*U*_iso_*/*U*_eq_	
Hg1	0.75260 (3)	0.03946 (3)	0.005310 (12)	0.02475 (11)	
Cl1	0.9924 (2)	0.02655 (19)	0.11378 (9)	0.0277 (3)	
Cl2	0.5237 (2)	0.01819 (19)	−0.10788 (9)	0.0288 (4)	
O1	0.7419 (7)	0.7485 (6)	0.0235 (3)	0.0439 (14)	
N1	0.7546 (6)	0.3191 (7)	0.0057 (3)	0.0248 (12)	
C2	0.7279 (8)	0.3824 (8)	0.0697 (4)	0.0264 (14)	
N3	0.7223 (7)	0.5265 (6)	0.0757 (3)	0.0245 (12)	
C4	0.7484 (8)	0.6201 (8)	0.0148 (4)	0.0286 (14)	
C4A	0.7784 (9)	0.5509 (7)	−0.0581 (4)	0.0238 (14)	
C5	0.8068 (9)	0.6256 (8)	−0.1257 (4)	0.0320 (15)	
H5A	0.8074	0.7237	−0.1240	0.038*	
C6	0.8339 (9)	0.5600 (8)	−0.1943 (4)	0.0321 (16)	
H6A	0.8510	0.6123	−0.2391	0.038*	
C7	0.8355 (9)	0.4115 (7)	−0.1964 (4)	0.0249 (13)	
H7A	0.8534	0.3659	−0.2434	0.030*	
C8	0.8111 (8)	0.3315 (7)	−0.1301 (4)	0.0249 (13)	
H8A	0.8143	0.2334	−0.1320	0.030*	
C8A	0.7813 (8)	0.4016 (7)	−0.0596 (3)	0.0193 (12)	
C9	0.6954 (10)	0.3200 (9)	0.1470 (4)	0.0374 (18)	
H9A	0.7924	0.2592	0.1716	0.045*	
H9B	0.5870	0.2654	0.1373	0.045*	
C10	0.6806 (13)	0.4488 (10)	0.2024 (5)	0.049 (2)	
H10A	0.5692	0.4460	0.2208	0.059*	
H10B	0.7757	0.4470	0.2499	0.059*	
C11	0.6914 (10)	0.5786 (8)	0.1545 (4)	0.0337 (17)	
H11A	0.5822	0.6319	0.1475	0.040*	
H11B	0.7880	0.6382	0.1810	0.040*	

### Atomic displacement parameters (Å^2^)

**Table:**

	*U*^11^	*U*^22^	*U*^33^	*U*^12^	*U*^13^	*U*^23^
Hg1	0.02197 (15)	0.03650 (19)	0.01675 (14)	0.00429 (11)	0.00622 (9)	0.00185 (9)
Cl1	0.0231 (7)	0.0420 (10)	0.0188 (7)	0.0049 (7)	0.0060 (5)	0.0053 (6)
Cl2	0.0229 (8)	0.0452 (10)	0.0189 (7)	−0.0008 (7)	0.0055 (6)	0.0021 (6)
O1	0.059 (4)	0.035 (3)	0.043 (3)	0.009 (3)	0.021 (3)	−0.011 (3)
N1	0.028 (3)	0.031 (3)	0.016 (2)	0.005 (2)	0.005 (2)	−0.0025 (19)
C2	0.024 (3)	0.036 (4)	0.019 (3)	0.009 (3)	0.005 (2)	0.003 (3)
N3	0.021 (3)	0.033 (3)	0.020 (2)	0.011 (2)	0.004 (2)	−0.002 (2)
C4	0.023 (3)	0.035 (4)	0.029 (3)	0.004 (3)	0.007 (2)	−0.004 (3)
C4A	0.021 (3)	0.026 (4)	0.023 (3)	0.008 (3)	0.000 (2)	−0.002 (2)
C5	0.035 (4)	0.031 (4)	0.029 (3)	−0.001 (3)	0.005 (3)	0.005 (3)
C6	0.033 (4)	0.041 (5)	0.024 (3)	0.001 (3)	0.008 (3)	0.009 (3)
C7	0.026 (3)	0.031 (4)	0.018 (3)	0.001 (3)	0.006 (2)	−0.001 (2)
C8	0.027 (3)	0.025 (3)	0.022 (3)	0.005 (3)	0.005 (2)	0.000 (2)
C8A	0.017 (3)	0.023 (3)	0.017 (2)	0.004 (2)	0.000 (2)	0.002 (2)
C9	0.044 (4)	0.052 (5)	0.021 (3)	0.014 (4)	0.019 (3)	0.004 (3)
C10	0.057 (5)	0.069 (7)	0.023 (3)	−0.021 (5)	0.014 (3)	−0.016 (4)
C11	0.030 (4)	0.047 (5)	0.027 (3)	0.010 (3)	0.011 (3)	−0.014 (3)

### Geometric parameters (Å, °)

**Table:**

Hg1—Cl1	2.3258 (16)	C5—C6	1.357 (10)
Hg1—Cl2	2.3302 (16)	C5—H5A	0.93
Hg1—Cl1^i^	3.1301 (16)	C6—C7	1.407 (10)
Hg1—Cl2^ii^	3.0416 (16)	C6—H6A	0.93
Hg1—O1^iii^	2.775 (6)	C7—C8	1.387 (9)
Hg1—N1	2.649 (6)	C7—H7A	0.93
O1—C4	1.227 (9)	C8—C8A	1.411 (8)
N1—C2	1.279 (8)	C8—H8A	0.93
N1—C8A	1.392 (8)	C9—C10	1.550 (11)
C2—N3	1.370 (9)	C9—H9A	0.97
C2—C9	1.488 (9)	C9—H9B	0.97
N3—C4	1.395 (9)	C10—C11	1.479 (12)
N3—C11	1.471 (8)	C10—H10A	0.97
C4—C4A	1.442 (9)	C10—H10B	0.97
C4A—C5	1.388 (9)	C11—H11A	0.97
C4A—C8A	1.415 (10)	C11—H11B	0.97
			
Cl1—Hg1—Cl2	171.44 (7)	C6—C7—H7A	119.3
Cl1—Hg1—N1	92.70 (11)	C7—C8—C8A	118.8 (6)
Cl2—Hg1—N1	95.27 (11)	C7—C8—H8A	120.6
C2—N1—C8A	117.9 (6)	C8A—C8—H8A	120.6
C2—N1—Hg1	118.0 (5)	N1—C8A—C8	117.8 (6)
C8A—N1—Hg1	124.1 (4)	N1—C8A—C4A	122.8 (5)
N1—C2—N3	122.7 (6)	C8—C8A—C4A	119.4 (5)
N1—C2—C9	128.6 (7)	C2—C9—C10	104.6 (7)
N3—C2—C9	108.6 (5)	C2—C9—H9A	110.8
C2—N3—C4	124.6 (5)	C10—C9—H9A	110.8
C2—N3—C11	114.4 (5)	C2—C9—H9B	110.8
C4—N3—C11	121.0 (6)	C10—C9—H9B	110.8
O1—C4—N3	121.9 (6)	H9A—C9—H9B	108.9
O1—C4—C4A	124.6 (7)	C11—C10—C9	108.1 (6)
N3—C4—C4A	113.6 (6)	C11—C10—H10A	110.1
C5—C4A—C8A	119.2 (6)	C9—C10—H10A	110.1
C5—C4A—C4	122.4 (7)	C11—C10—H10B	110.1
C8A—C4A—C4	118.3 (6)	C9—C10—H10B	110.1
C6—C5—C4A	122.1 (7)	H10A—C10—H10B	108.4
C6—C5—H5A	118.9	N3—C11—C10	104.1 (6)
C4A—C5—H5A	118.9	N3—C11—H11A	110.9
C5—C6—C7	118.9 (6)	C10—C11—H11A	110.9
C5—C6—H6A	120.6	N3—C11—H11B	110.9
C7—C6—H6A	120.6	C10—C11—H11B	110.9
C8—C7—C6	121.4 (6)	H11A—C11—H11B	109.0
C8—C7—H7A	119.3		

Symmetry codes: (i) −*x*+2, −*y*, −*z*; (ii) −*x*+1, −*y*, −*z*; (iii) *x*, *y*−1, *z*.

### Hydrogen-bond geometry (Å, °)

**Table:**

*D*—H···*A*	*D*—H	H···*A*	*D*···*A*	*D*—H···*A*
C5—H5A···Cl1^iv^	0.93	2.81	3.630 (8)	147.
C10—H10B···Cl2^v^	0.97	2.76	3.724 (9)	171.

Symmetry codes: (iv) −*x*+2, −*y*+1, −*z*; (v) *x*+1/2, −*y*+1/2, *z*+1/2.
